# What Are the Key Determinants of Sustainable Organizational Performance? A PRISMA-Based Systematic Review

**DOI:** 10.12688/f1000research.168678.1

**Published:** 2025-10-31

**Authors:** Suparwadi Suparwadi, Mochammad Al Musadieq, Muhammad Faisal Riza, Benny Hutahayan

**Affiliations:** 1 Brawijaya University, Malang, East Java, Indonesia

**Keywords:** Organizational Performance; Systematic Literature Review; PRISMA; Bibliometrics; Knowledge Management.

## Abstract

This study aims to explore influential and relevant factors affecting organizational performance. A Systematic Literature Review (SLR) approach was employed to address this objective. The systematic literature review in this study adheres to the Preferred Reporting Items for Systematic Reviews and Meta-Analyses (PRISMA) guidelines. PRISMA was chosen because it provides a comprehensive and relevant framework for reviewing literature related to the research topic. Additionally, it offers a systematic protocol that enhances the quality and clarity of research reporting. Data were obtained from the Scopus database, covering publications from 2019 to 2024. The articles analyzed were selected based on predefined criteria. The findings reveal that the key determinants of organizational performance include knowledge management, sustainable development, performance assessment, supply chain management, and human resource management. These findings offer practical implications for organizations seeking to enhance their performance. The novelty of this study lies in its exclusive focus on uncovering the complex factors that influence organizational performance using the SLR method grounded in the PRISMA approach. By adopting this method, the study aims to provide deeper insights and nuanced understanding that goes beyond general knowledge, thereby enriching academic literature with a fresh perspective on the multifaceted factors shaping organizational success.

## 1. Introduction

Organizational performance is a crucial aspect of business and management. ^1, 2, 3^ The ability of an organization to achieve its goals, meet stakeholder expectations, and continue to adapt and develop in an ever-changing environment is essential. ^4– 6^ According to, ^7^ performance is done or not done by employees. Performance influences how much employees contribute to the organization. Robbins ^8^ explains that performance is human output measured by Productivity, absenteeism, turnover, citizenship, and satisfaction. Therefore, a deep understanding of the factors that influence organizational performance becomes very relevant and important to study.

As business dynamics evolve and technology continues to advance, the study of factors that influence organizational performance also evolves and grows. ^9– 11^ Various studies have been conducted to identify these factors, but there is still room for further investigation. Because each organization has its context, the factors influencing organizational performance can vary widely. ^12^ This research aims to investigate the determinants of organizational performance in more depth. With a better understanding of these factors, organizations can take more appropriate steps to improve their performance. Additionally, this research will contribute to the scientific literature in management and business by providing valuable insights into the interrelated factors that influence organizational performance. This research also becomes relevant considering the increasingly tight business competition and pressure to achieve optimal performance.

Previous research, ^13^ shows a positive and significant influence of product and process innovation on sustainable innovation, and sustainable innovation has a substantial impact on environmental Performance and Organizational Performance. Meanwhile, The participation of low-level managers in setting corporate performance targets has a positive impact on organizational performance. ^14^ The research results by Schiuma et al., ^15^ show that digital leadership has both direct and indirect positive impacts on organizational performance. In addition, digital culture and employees’ digital capabilities partially mediate the relationship between digital leadership and Organizational Performance.

Research that uses a systematic literature review for the topic of organizational performance has actually been conducted by several researchers. ^13– 15^ However, this study focuses more on determining what variables affect organizational performance. The systematic literature review conducted by Kurniawati & Sulaeman ^16^ examines the relationship between human resource management practices and organizational performance. There is another SLR study regarding the organization conducted by. ^17^ Performance measurement systems are crucial for evaluating organizational effectiveness, with various measures identified to aid decision-makers. ^18^ Organizational learning has also been linked to improved performance, particularly in Asian contexts. ^19^ Another SLR study in the organization field has been conducted by. ^20^ Human resource managers play a vital role in implementing innovative strategies for employee empowerment and creating a positive organizational culture. ^21^ The studies emphasize the importance of adapting to changing environments and leveraging human resources to enhance organizational performance.

Despite significant advancements in understanding organizational performance, several critical gaps remain unaddressed. A notable omission is the exploration of behavioral and in-dividual-level dynamics. Concepts such as employee engagement and leadership are frequently discussed in the literature, ^14, 15^ the PRISMA and bibliometric analyses will delve deeper into how these theories are explained. This focus neglects the complex ways in which individual behaviors, attitudes, and motivations influence collective performance outcomes. The reviewed studies often center on specific industries or geographic regions, leaving a gap in understanding how organizational performance drivers differ across diverse cultural and economic contexts. Although some studies focus on performance within specific industries, the generalization of findings limits their applicability to niche sectors with unique operational challenges and per-formance metrics. Addressing these gaps is essential for developing a comprehensive and nu-anced understanding of organizational performance.

This paper employs a Systematic Literature Review (SLR) guided by the PRISMA (Preferred Reporting Items for Systematic Reviews and Meta-Analyses) framework to explore the key determinants of organizational performance. ^22, 23^ The PRISMA methodology offers a structured and transparent approach to identifying, selecting, and synthesizing relevant studies. ^24^ It enhances methodological rigor by minimizing selection bias and ensuring reproducibility through four main phases: identification, screening, eligibility, and inclusion. ^25^ By emphasizing transparency, consistency, and replicability, the PRISMA-based SLR also helps identify research gaps and offers direction for future studies. ^24^

## 2. Literature review

### 2.1 Systematic literature review

A Systematic Literature Review (SLR) is a structured research method designed to collect and examine various study results related to a specific topic as a basis for decision-making. ^26^ The data used comes from original research published in journal articles, particularly at the national level. ^27^ Data collection is carried out through various indexed electronic databases, such as Google Scholar, Semantic Scholar, ERIC, and direct access to national journals. ^28^ The implementation of SLR has various objectives, including identifying, reviewing, evaluating, and interpreting the entire research study in a field of phenomena relevant to the research questions that have been formulated. ^23^ In addition, SLR is often used to determine the direction of research, both in the preparation of theses and dissertations, and is an important element in the design of research proposals. ^22^

According to Khan et al., ^29^ there are five stages in carrying out a Systematic Literature Review. This structured method provides a comprehensive and rigorous framework for identifying, evaluating, and synthesizing relevant studies on organizational performance. In practice, the application of SLR not only ensures transparency and repeatability of the research process but also minimizes potential bias in the selection and synthesis of studies. ^30^ With a systematic approach, researchers can build a strong theoretical foundation, identify research gaps, and generate insights that contribute to the development of science and its practical implications. ^31^ This makes SLR particularly valuable in management and organizational studies, where evidence-based recommendations are needed to support appropriate strategies and policies. ^32^

### 2.2 Organizational performance

Performance is something that employees either do or do not do. Performance influences how much employees contribute to the organization. ^7^ Meanwhile, according to Robbins, ^8^ performance is human output measured by Productivity, absenteeism, turnover, citizenship, and satisfaction. Organizational performance is a complex concept influenced by various factors. Leadership plays a crucial role, with effective leaders creating cooperative work environments and engaging teams emotionally. ^33^ Based on the explanation above, organizational performance measures how an organization achieves its goals by creating value, enhancing efficiency, and improving effectiveness in its operations. Organizational performance is one of the important factors and variables in improving organizational performance; this concept is often found in the organizational development process.

According to Broderick, ^34^ several dimensions are determined, including the organizational performance dimension, namely helping business success as the dependent variable. These dimensions are known as follows.

a. Role Sets
b. Role Scripts
c. Role Congruence
d. Role Performance
e. Role Expansion
f. Role Discrepancy
g. Role Conflict

Organizational performance measurement has evolved beyond traditional financial metrics to include non-financial factors and stakeholder expectations. ^35^ Recent studies emphasize the need for a comprehensive approach, integrating both financial and non-financial indicators to reflect organizational performance more accurately. ^36^ Key dimensions of performance measurement include quality, customer satisfaction, innovation, and stakeholder perceptions. ^37^ A study on Kenyan contractors found that quality of products and client satisfaction were the highest-performing dimensions, while profitability and employee satisfaction scored lowest. ^38^ To effectively measure organizational performance, researchers should review commonly used metrics, classify them into financial and non-financial or objective and subjective measures, and then integrate these indicators. ^36^ This multi-dimensional approach allows for a more comprehensive evaluation of organizational performance, addressing the complexities of modern business environments.

## 3. Research methods

In this study, we utilized a systematic literature review approach to identify and evaluate the key factors influencing organizational performance. The research was conducted in May 2025 and followed the PRISMA (Preferred Reporting Items for Systematic Reviews and Meta-Analyses) guidelines. ^25^ PRISMA was developed to enhance the transparency and rigor of systematic reviews and meta-analyses. ^39^ It was chosen for this study because it provides a comprehensive and relevant framework for reviewing literature related to the research topic. ^23^ Additionally, PRISMA offers a systematic protocol that improves the quality and clarity of research reporting. ^40^ The PRISMA approach facilitates a thorough analysis of existing literature, identification of research gaps, and formulation of future research directions. ^41^ The most recent updates to the PRISMA guidelines further strengthen reporting standards by including elements such as risk of bias assessments and the declaration of competing interests. ^25^

### 3.1 Source

This study used Scopus as the primary data source for selecting articles to be analyzed. Scopus was chosen because it is one of the largest and most reputable scientific databases currently available. ^42^ By using Scopus, the findings of this study are expected to be highly relevant and contribute to the development and reinforcement of existing theories. ^24^ In addition, Scopus provides comprehensive coverage across multidisciplinary fields, ensuring that the selected articles represent a diverse and credible body of academic research.

### 3.2 Inclusion and exclusion criteria

To ensure that the selected articles aligned with the objectives of this research, we applied eligibility criteria. This step was crucial to guarantee the relevance and quality of the data included in the analysis. The criteria used in this study are as follows:

1. Articles must focus on the organizational performance of companies.
2. Articles must be final publications that have undergone the peer-review process.
3. Articles must be international and written in English.
4. Articles must be published within the last five years (2019–2024).
5. Articles must be complete, containing an abstract, background, methodology, results and discussion, and conclusion.
6. Articles may use qualitative, quantitative, or mixed-method approaches.

By applying these criteria, only articles that are relevant and aligned with the research objectives were selected for further analysis. Articles that did not meet the criteria were excluded from the review process. Meanwhile, this study excluded (exclusion criteria) articles that did not directly discuss organizational performance, were incomplete (lacking an abstract or other essential sections), or were preprint articles.

### 3.3 Data collection process

The process began by determining the appropriate search keywords, with “Organizational Performance” selected as the primary terms. The search was then carried out on Scopus using the query TITLE (“Organizational Performance”), which targeted article titles, abstracts, and keywords. This approach allowed the researchers to comprehensively identify literature relevant to the topic while maintaining control over the selection process through careful manual screening and evaluation.

### 3.4 Data item selection and synthesis

**Figure 1.**
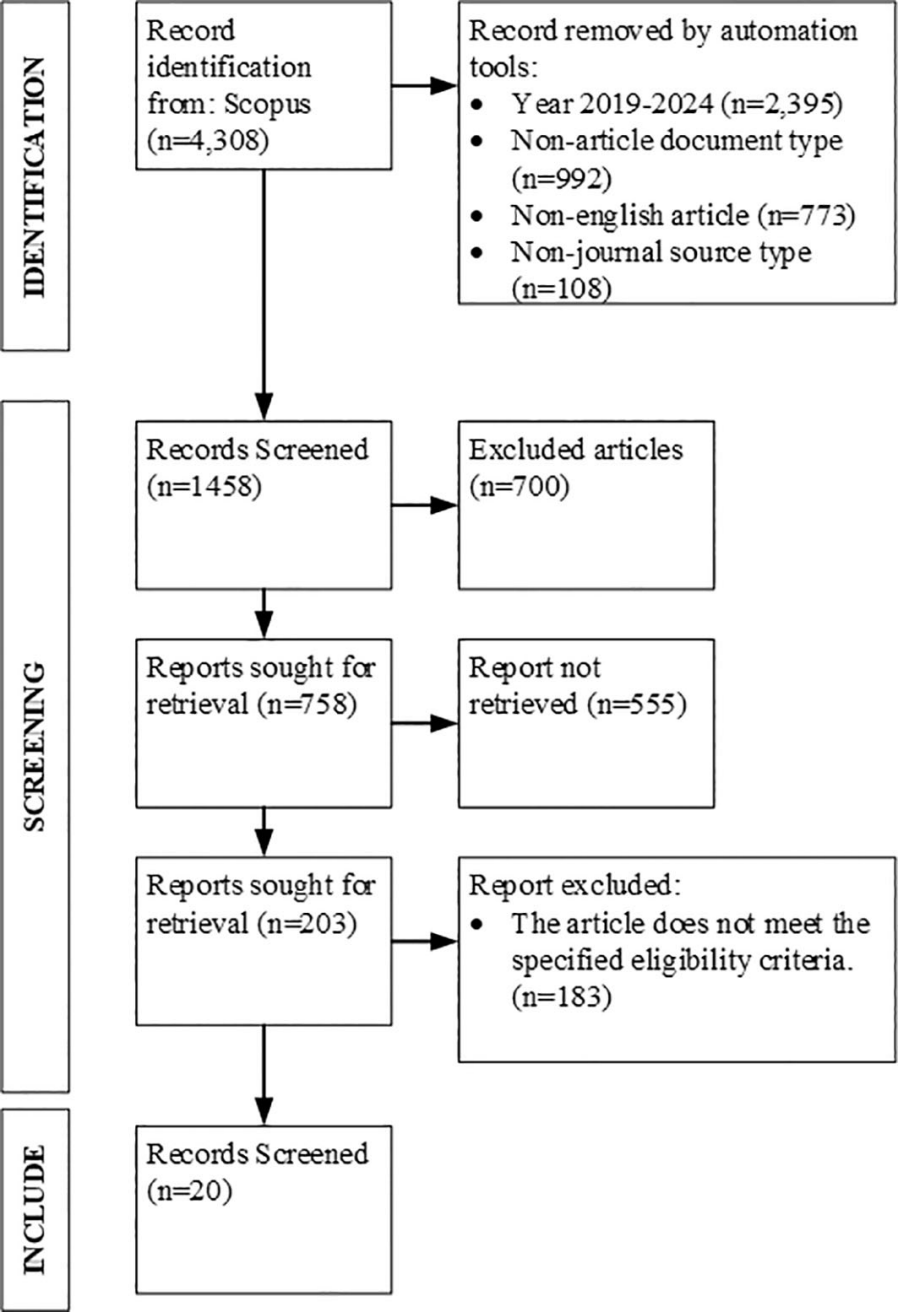
PRISMA flow diagram.

This review was conducted in accordance with the guidelines set by the Preferred Reporting Items for Systematic Reviews and Meta-Analyses (PRISMA). ^39^ The process was organized into multiple essential steps to guarantee a thorough and meticulous analysis. To find out the flow of article selection in this research, a PRISMA flow diagram was created which is presented in Figure 1 below.

The initial search produced a substantial number of studies, which were filtered through a two-step screening process. First, titles and abstracts were reviewed to determine their relevance. Articles that appeared to satisfy the inclusion criteria proceeded to a full-text review. In this stage, studies were evaluated based on methodological quality and alignment with the research question. Only studies meeting all criteria were included in the final synthesis.

Data extraction targeted key elements, such as research objectives, methodologies, findings, and theoretical contributions, which were systematically documented in a data extraction form. The collected data were then synthesized narratively, allowing for the identification of recurring patterns, themes, and gaps within the literature. To ensure thoroughness, backward and forward citation tracking was utilized. This process involved reviewing references of selected studies (backward citation) and identifying more recent studies that referenced them (forward citation). This research was conducted using the Scopus database combined with manual analysis to reduce potential bias and ensure a more accurate selection of relevant studies. In addition, to minimize the risk of inter-author bias during the review process, more than one researcher independently evaluated each article, and the results were compared and agreed upon through joint discussion.

## 4. Result

Based on Figure 1, the initial search yielded a total of 4,308 articles. The next step involved the initial screening (identification), in which the articles were selected based on the predetermined publication period (2019–2024). Articles that were still in the press, non-English, or from non-journal sources were excluded. This step was necessary to ensure that only articles meeting the inclusion criteria and relevant to the research were analyzed. As a result of this process, a total of 1,458 articles remained.

The next step was the second screening phase. This process was conducted using the filtering features available in Scopus. Filters were applied in accordance with the predetermined criteria, such as requiring the article to discuss “organizational performance” by including this keyword and meeting other eligibility standards. After this filtering process, 758 articles were retained.

The following step was to assess the quality of the articles. Incomplete articles, including manuscripts lacking identifiable authors or credible sources, were excluded. This step was carried out manually by reading the titles and abstracts of the articles that passed the previous stage. As a result, 203 valid articles were identified.

The final stage involved a full content review of each article, which was also conducted manually. To minimize errors and potential bias, the study used Microsoft Word and Excel to record all relevant information from each article. After this comprehensive screening process, 20 articles were found to be valid and aligned with the objectives of this study. These 20 articles are summarized in Table 1.

**Table 1.** Research articles related to organizational performance.

No	Authors	Years	Title	Purpose	Key finding
1	George et al. ^43^	2019	Does strategic planning improve organizational performance? A meta-analysis.	To analyze the impact of strategic planning on organizational performance through meta-analysis.	Strategic planning positively, moderately, and significantly affect organizational performance, especially when measured as effective and conducted formally.
2	Kurdi et al. ^44^	2020	Employee retention and organizational performance: Evidence from banking industry.	To identify the factors affecting employee retention and their impact on organizational performance in Jordan's banking sector.	Economic, psychological, affiliation and self-actualization factors influence retention and affect organizational performance.
3	Singh & Misra ^45^	2021	Linking corporate social responsibility (CSR) and organizational performance: The moderating effect of corporate reputation.	To examine the relationship between corporate social responsibility (CSR) and organizational performance, with corporate reputation as a moderator.	External CSR has a positive impact on performance and is stronger in highly reputable companies.
4	Lee et al. ^46^	2022	The effect of digital supply chain on organizational performance: An empirical study in Malaysian manufacturing industry.	To assess the impact of digital supply chains on supply chain and organizational performance in Malaysia's manufacturing industry.	Digital supply chains significantly enhance performance; most hypotheses were supported, but some were not.
5	Kumar et al. ^47^	2023	Influence of data-driven supply chain quality management on organizational performance: evidence from the retail industry.	To examine the relationship between data-driven supply chain quality management practices and retail performance in India.	"Customer focus" and "employee relations" have the most significant impact on performance.
6	Kadir et al. ^48^	2024	The relationships amongst career patterns, neutrality and organizational performance: the case of local government organizations in South Konawe District, Indonesia	To analyze the relationship between career patterns, civil servant neutrality, and organizational performance in Indonesian local governments.	Career patterns affect neutrality and performance; promotions have the most substantial effect, and demotions are the weakest.
7	Nu Graha et al. ^49^	2019	“The role of knowledge management in organizational performance: Case study of University of Malang, Indonesia”	To test the impact of HRM, organizational culture, and knowledge management on organizational performance.	Culture and knowledge management significantly impact performance; HRM has only an indirect effect.
8	Shen et al. ^50^	2022	The Mechanism of Digital Environment Influencing Organizational Performance: An Empirical Analysis Based on Construction Data	To explain how organizations can enhance their performance in a digital environment.	Organizational behavior aligned with the digital environment significantly improves performance.
9	Zhang et al. ^51^	2022	Exploring the Mutual Nexus of Social Capital, Social Innovations and Organizational Performance	To analyze the role of organizational and social innovation as mediators between social capital and performance.	Innovation significantly mediates the relationship; no direct link was found between social capital and performance.
10	Nguyen et al. ^52^	2023	The moderating effect of perceived environmental uncertainty and task uncertainty on the relationship between performance management system practices and organizational performance: evidence from Vietnam	To analyze the effect of environmental and task uncertainty on the effectiveness of performance management systems in Vietnam.	Some PMS practices are effective depending on the level of uncertainty faced.
11	Lee et al. ^53^	2019	Outsourcing and Organizational Performance: The Employee Perspective	To analyze the impact of outsourcing on organizational performance and federal employee job satisfaction in the U.S.	Outsourcing negatively impacts performance through reduced job satisfaction.
12	Anwarul Islam et al. ^54^	2024	Revisiting the impact of entrepreneurial orientation on SMEs' organizational performance	To measure the effect of entrepreneurial orientation on SME performance in Bangladesh.	All dimensions have a positive impact, with proactiveness being the most dominant.
13	Fuzi et al. ^55^	2022	Sustainability Management Accounting and Organizational Performance: The Mediating Role of Environmental Management System.	To explore the relationship between sustainable management accounting, environmental management systems, and organizational performance.	Both factors have positive effects, and the environmental system mediates the impact of accounting on performance.
14	Hu et al. ^56^	2019	Past performance, organizational aspiration, and organizational performance: The moderating effect of environmental jolts.	To test the influence of past performance and aspiration on current performance under environmental shocks.	The influence varies depending on the level of environmental shock.
15	Bhatti et al. ^57^	2020	Organizational capabilities mediates between organizational culture, entrepreneurial orientation, and organizational performance of smes in pakistan.	To examine the role of organizational capabilities as a mediator between organizational culture, entrepreneurial orientation, and SME performance.	Organizational capabilities significantly mediate both relationships.
16	Abu-Mahfouz et al. ^58^	2023	Sustainable human resource management practices in organizational performance: The mediating impacts of knowledge management and work engagement.	To analyze the impact of sustainable HRM on performance through knowledge management and work engagement.	Significant effect exists through a dual mediation path.
17	Nawaz Khan et al. ^59^	2019	The mediating role of innovation between corporate governance and organizational performance: Moderating role of innovative culture in Pakistan textile sector.	To analyze the effect of corporate governance elements on organizational performance with innovation as a mediator.	Board size and diversity influence performance; innovation mediates, and an innovative culture moderates the relationship.
18	Rahmat et al. ^60^	2024	Evaluating the Role of Open Innovation and Circular Economy in Enhancing Organizational Performance: Insights from Batik Small and Medium Enterprises in Banyuwangi, Indonesia	To examine the impact of open innovation and circular economy principles on the performance of batik SMEs in Indonesia.	Resource optimization and innovation networks are more effective than waste management.
19	Habeeb and Eyupglu ^61^	2024	Strategic Planning, Transformational Leadership and Organization Performance: Driving Forces for Sustainability in Higher Education in Nigeria.	To assess the effect of strategic planning on transformational leadership and university performance in Nigeria.	Strategic planning improves transformational leadership and organizational performance.
20	Lee et al. ^62^	2023	Effects of College Faculty Members’ Entrepreneurial Orientation on Organizational Performance: Case of South Korea.	To examine the effect of lecturers' entrepreneurial orientation on organizational performance in South Korean universities.	Innovation, proactiveness, and risk-taking by lecturers positively impact competitiveness and organizational commitment.

Table 1 presents a synthesis of 20 empirical studies exploring various organizational performance determinants across sectors and countries. The findings indicate that strategic planning, employee-related factors, innovation, digital transformation, and governance practices significantly influence organizational performance. Strategic planning enhances effectiveness and organizational outcomes, particularly when conducted formally and aligned with transformational leadership. Employee factors such as retention, job satisfaction, and psychological engagement also play a vital role in shaping performance. Innovation—whether driven by entrepreneurial orientation, social capital, or governance structures—emerges as a recurring mediator that strengthens organizational capabilities and competitiveness.

Additionally, the integration of digital technologies and data-driven management systems contributes positively to performance, especially in dynamic and uncertain environments. Studies from developing economies further highlight the importance of contextual adaptability, sustainable practices, and the role of knowledge management. Overall, the literature underscores that improving organizational performance requires a holistic approach that combines strategic, human, technological, and cultural dimensions.

**Figure 2.**
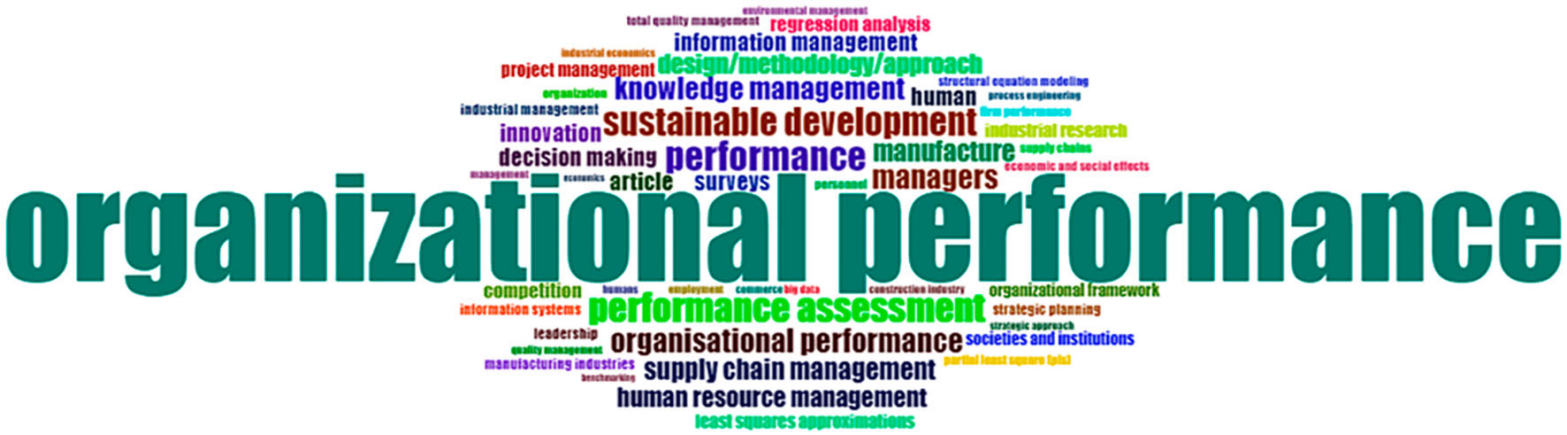
Dominant keywords.

This study also identifies the most frequently used keywords in the analyzed articles. This study was done to explore what topics are commonly associated with “organizational performance.” Understanding these keywords helps broaden insights into the various dimensions and contexts of organizational performance. Figure 2 below illustrates the relevant keywords found in the articles related to organizational performance.

The most dominant words in research with the theme of Organizational Performance will be displayed in word cloud form. Figure 2 or known as word cloud is a visual representation of words frequently appearing in a data set of researched articles using keywords related to organizational performance. The word cloud will display these words in varying sizes according to the frequency with which they appear. The placement of words in the word cloud is random, but the most dominant terms will be placed in the middle and have a large size. In this research, word clouds were created based on the analysis of article titles. This analysis found that the most dominant words were related to knowledge management, managers, manufacturing, organizational performance, organizational performance, performance, performance assessment, supply chain management, and sustainable development. Most of the research related to Organizational Performance is closely associated with the issue of knowledge management. This shows that knowledge management will be an important determining factor for achieving sustainable organizational performance in future research.

In addition, this study also identifies the most frequently studied subject areas in articles discussing organizational performance. The purpose of this analysis is to provide insight for future research regarding the most relevant subject areas within this topic. The figure below presents the subject areas that are most commonly addressed in studies related to organizational performance.

**Figure 3.**
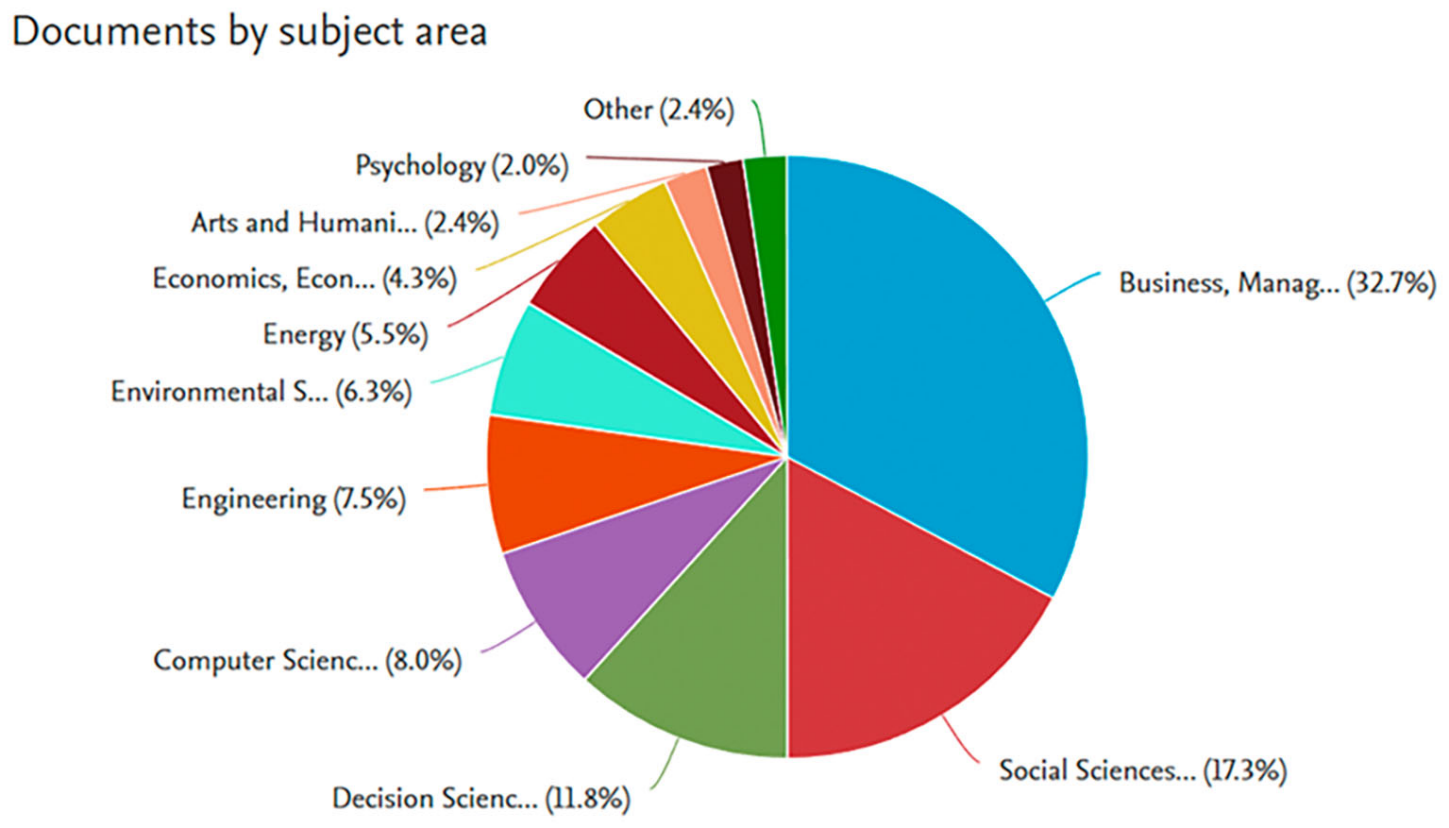
Publication by subject area.

The pie chart shown in Figure 3 highlights the multidisciplinary nature of job performance research, with the majority of studies conducted in Business and Management (32.7%), reflecting the critical role of job performance in organizational and corporate settings. Social Sciences (17.3%) and Decision Sciences (11.8%) follow, emphasizing the importance of understanding performance in societal contexts and decision-making processes. Disciplines like Computer Science (8.0%), Engineering (7.5%), and Environmental Sciences (6.3%) also contribute significantly, exploring the influence of technology, technical processes, and environmental factors on performance. Smaller shares are observed in Economics (4.3%), Energy (5.5%), Psychology (2.0%), and Arts and Humanities (2.4%), indicating niche yet meaningful contributions. While Business dominates, the chart illustrates how job performance research spans various fields, showcasing its relevance across diverse academic and practical domains.

## 5. Discussion

The findings from Table 1 reveal a multifaceted and interconnected set of variables that significantly influence organizational performance. These variables span diverse domains, including human resource management, technological adoption, sustainability, and performance management systems. The systematic analysis uncovers both direct and mediated relationships, demonstrating how these factors interact to shape job performance in organizational contexts.

Human resource management and organizational culture emerge as central themes. Multiple studies highlight that sustainable HR practices play a critical role in fostering knowledge management and employee engagement, which in turn enhance organizational performance. For example, Abu-Mahfouz et al. ^58^ Illustrate how sustainable HR management can simultaneously improve work engagement and facilitate effective knowledge-sharing practices. Furthermore, Nu Graha et al. ^49^ emphasize the partial mediation role of knowledge management between HR practices and performance outcomes, underscoring its pivotal position in driving productivity and efficiency.

Technological advancements and digital tools are equally influential. The integration of digital environments, such as digital supply chain management and sustainability-oriented accounting systems, has been shown to boost organizational performance through increased efficiency and alignment with market demands. Shen et al. ^50^ propose a structural model linking the digital environment to organizational behavior and performance, demonstrating the importance of technological adaptation in modern organizations. Similarly, Lee et al. ^46^ and Fuzi et al. ^55^ highlight the transformative potential of digital tools in reshaping supply chains and management accounting systems to optimize performance outcomes.

Another critical dimension is the role of innovation and its interplay with organizational culture. Social and organizational innovations act as mediators between social capital and performance. ^51^ This suggests that fostering a culture of innovation is essential for organizations aiming to enhance adaptability and competitive advantage. Furthermore, leadership aspirations and historical performance trends, as studied by Hu et al., ^56^ are shown to have varying impacts on performance based on environmental stability, highlighting the nuanced role of leadership in dynamic contexts.

The findings also emphasize the importance of effective performance management systems, especially in uncertain environments. Nguyen et al. ^52^ illustrate that involving lower-level managers in setting performance targets and decentralizing decision-making enhances organizational outcomes, particularly in the face of environmental and task-related uncertainties. This reflects the growing recognition of participatory and adaptive approaches in performance management.

Finally, sustainability and environmental considerations significantly shape organizational performance. Studies reveal that implementing sustainability-focused practices, such as environmental management systems, not only aligns organizations with societal expectations but also strengthens employee engagement and operational resilience. The emphasis on sustainability, as reflected in several articles, signals a paradigm shift where long-term environmental goals complement immediate performance objectives.

In summary, the analysis highlights a complex interplay of variables influencing organizational performance, including human resource practices, knowledge management, technological advancements, innovation, and sustainability. These factors collectively inform strategies for enhancing job performance and organizational outcomes, providing a comprehensive foundation for future research and practical application.

Based on the SLR using Bibliometrics and PRISMA, several variables that are most significant in influencing purchasing decisions have been identified. Some of these variables are as follows.

**Figure 4.**
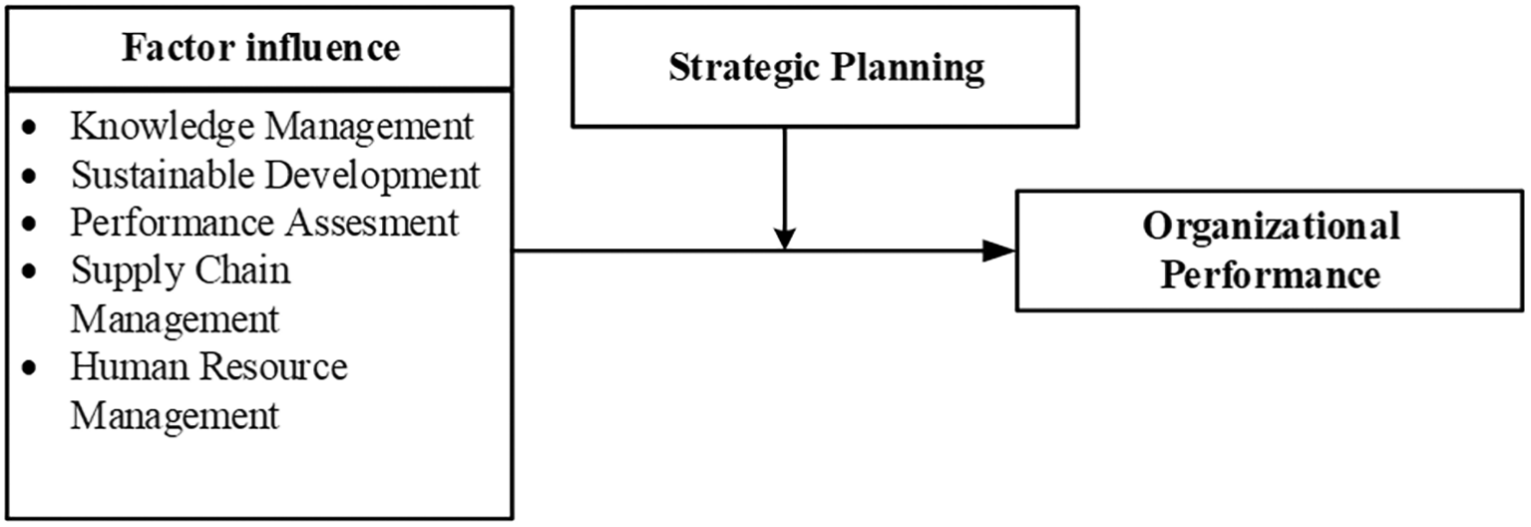
Publication by subject area.

Several topics have great potential for further research in the context of organizational performance. The visualization of the results, presented in several figures, provides valuable information for researchers to identify issues that have the potential for new contributions in the field of Organizational Performance. The analysis results regarding the factors that influence organizational performance, as shown in the picture above, include knowledge management, sustainable development, performance assessment, supply chain management, and human resource management (see Figure 4).

The findings from the PRISMA and bibliometric analyses align with and expand upon the definitions, concepts, and indicators of organizational performance discussed in the literature review. Organizational performance has been broadly defined as the ability of an organization to achieve its goals, create value, and maintain efficiency and effectiveness in operations. Authors such as ^7^ describe performance as the contributions employees make to their organizations, while ^8^ views it as a multidimensional measure encompassing productivity, absenteeism, turnover, citizenship, and satisfaction. These definitions emphasize the holistic nature of performance, which includes both financial and non-financial dimensions. The PRISMA findings reflect this multidimensional view by identifying factors such as knowledge management, sustainability, and digital transformation as critical contributors to organizational performance. For instance, the recognition of knowledge management as a key determinant underscores its role in fostering innovation, enhancing operational efficiency, and driving overall success.

Several indicators of organizational performance are emphasized in previous research, ranging from traditional financial metrics to modern, non-financial measures. Gutterman ^35^ highlights the integration of financial and non-financial indicators, including quality, customer satisfaction, and innovation. Similarly, Simon et al. ^38^ emphasize that product quality and client satisfaction often surpass profitability and employee satisfaction as significant performance dimensions. Barradas Martinez et al. ^37^ advocate for a comprehensive evaluation approach that incorporates stakeholder perceptions and operational efficiency. These concepts align closely with the PRISMA and bibliometric results, which emphasize the role of performance management systems and digital tools in achieving organizational success. For instance, Shen et al. ^50^ demonstrate how digital transformation directly impacts operational efficiency and quality, indicators prominently discussed in earlier studies. Similarly, the focus on sustainability practices, such as sustainability management accounting systems, ^55^ reflects a growing emphasis on environmental performance as an essential organizational metric.

Previous research has also highlighted specific themes such as knowledge management, leadership, organizational culture, and sustainability. Nu Graha et al. ^49^ underscore knowledge management’s mediating role between HR practices and performance, emphasizing its ability to facilitate organizational learning and drive sustainable competitive advantage. This is strongly validated by the PRISMA findings, which highlight the prominence of knowledge management as a recurring theme. Moreover, leadership and organizational culture are identified as critical drivers of innovation and engagement in studies like those by. ^15, 57^ However, while leadership was not a dominant theme in the PRISMA findings, the bibliometric analysis underscores the importance of organizational culture, with Abu-Mahfouz et al. ^58^ showing how sustainable HR practices foster employee engagement and performance.

Sustainability and environmental considerations also feature prominently in both the literature and the PRISMA results. Fuzi et al. ^55^ discuss how environmental management systems act as mediators in enhancing organizational performance, demonstrating that sustainability is not only an ethical imperative but also a practical strategy for achieving performance goals. The PRISMA findings corroborate this by emphasizing the growing importance of integrating sustainability into organizational practices.

While the PRISMA and bibliometric findings validate many of the definitions, concepts, and indicators presented in the literature, specific gaps remain. The PRISMA analysis primarily focuses on organizational-level factors and metrics, leaving individual-level dynamics, such as employee motivation and psychological engagement, less explored. These dimensions, emphasized in studies like Nguyen and Ngo, ^52^ highlight the need for further investigation into how individual behaviours influence organizational outcomes. Additionally, the PRISMA findings provide broad insights but lack the granularity of sector-specific analyses, such as those by Simon et al., ^38^ which offer more tailored performance indicators for specific industries.

## 6. Conclusion and implications

The findings highlight critical drivers of organizational performance, such as knowledge management, sustainability, and digital transformation, while revealing significant literature gaps. Addressing these gaps through longitudinal studies, sector-specific research, and instruments for individual-level dynamics and technological readiness will advance the field. The study underscores the importance of integrating modern tools and strategies with traditional organizational performance management practices. Future studies can provide deeper insights and actionable frameworks to enhance organizational performance by addressing the identified limitations and pursuing the recommended research directions.

The findings from this study offer valuable implications for organizations aiming to enhance their performance. A primary takeaway is the importance of knowledge management as a driver of organizational success. Organizations should prioritize systems and practices that facilitate knowledge sharing, continuous learning, and innovation. Furthermore, the integration of sustainability practices into core operations is not only a societal expectation but also a strategic advantage. Sustainability-focused initiatives can enhance long-term resilience and improve stakeholder relationships, as highlighted in studies like. ^55^

The increasing role of digital transformation presents another crucial implication. Organizations must invest in digital tools and technologies to optimize processes, improve decision-making, and adapt to dynamic market conditions. Shen et al. ^50^ emphasize the transformative impact of digital environments on organizational performance, suggesting that such investments are no longer optional but essential. Finally, effective leadership development is vital. Training programs that emphasize adaptability, inclusivity, and innovation are necessary to navigate the complexities of modern organizational challenges. Organizations should also foster decentralized decision-making to improve adaptability, particularly in uncertain environments.

## 7. Limitations and future research directions

This study has several limitations that need to be considered when interpreting its findings. One limitation lies in the scope of data sources. By relying primarily on Scopus and similar databases, the study may have excluded relevant studies published in non-indexed or regional journals, particularly those from emerging economies where organizational performance research might offer unique perspectives. Additionally, the focus on recent publications (2019–2024) ensures contemporary relevance but potentially overlooks foundational works that have shaped the field. Incorporating older studies could provide a more robust understanding of the evolution of key concepts.

Another limitation is the generalizability of the findings. Given the focus on specific industries and regions, the results may not fully capture the nuances of organizational performance across diverse contexts. This is particularly pertinent when considering cultural and economic differences that influence performance drivers. Lastly, the PRISMA and bibliometric methodologies, while systematic, may inherently favor certain types of studies, potentially overlooking qualitative research that could provide deeper insights into the relational and contextual aspects of performance.

Future research should aim to address these gaps by adopting more integrative and nuanced approaches. Longitudinal studies are needed to explore causal relationships and track the evolution of performance drivers over time. These studies would provide a deeper understanding of how variables such as knowledge management, sustainability, and digital transformation interact in dynamic contexts. Additionally, the development of behavioral instruments such as engagement surveys and psychological assessments would allow researchers to capture individual-level contributions to organizational performance. Tools that assess technological readiness, such as AI-readiness indices, are also essential for understanding how organizations can effectively integrate emerging technologies.

The inclusion of cross-cultural frameworks in future research is critical for examining how cultural differences mediate the effectiveness of performance strategies. Models such as Hofstede’s cultural dimensions could be employed to investigate these variations. Furthermore, sector-specific analyses are necessary to provide tailored insights for industries with unique operational challenges. Detailed case studies or industry-specific frameworks could fill this gap, offering practical guidance for organizations within these sectors. Expanding the scope of data sources to include grey literature and regional studies will also ensure a more comprehensive representation of organizational performance research. By addressing these directions, future studies can offer richer insights and more actionable recommendations for both academia and practice.

## Ethical approval statement

Not applicable.

## Data Availability

No data associated with this article. Repository name: *Data SLR “What Are the Key Determinants of Sustainable Organizational Performance? A PRISMA-Based Systematic Review”.* https://doi.org/10.6084/m9.figshare.29661842. ^63^ This project contains the following extended data: [Data SLR] (Collection of article data used for literature review). Figshare: PRISMA checklist for “What Are the Key Determinants of Sustainable Organizational Performance? A PRISMA-Based Systematic Review”. https://doi.org/10.6084/m9.figshare.29661842. ^63^ Data are available under the terms of the Creative Commons Attribution 4.0 International license (CC-BY 4.0).
