# Supplementary material for: What Are the Key Determinants of Sustainable Organizational Performance? A PRISMA-Based Systematic Review
Source: F1000Res. 2025 Oct 31;14:1189. [Version 1] doi: 10.12688/f1000research.168678.1 (PMC12715398; doi:10.12688/f1000research.168678.1)
Supplement: Peer review history

# Reviewer report: Reviewer response for version 1

**DOI:** 10.5256/f1000research.185871.r429248

**Published:** 2025-12-18

**Authors:** Belayneh Yitayew Kassa

**Affiliations:** 1 Arba Minch University, Arba Minch, Ethiopia

Thank you for the opportunity to review this manuscript. The study addresses a timely and significant topic at the key determinants of sustainable organizational performance. However, the manuscript will benefit greatly from a better aesthetic appearance, and for this reason, I offer many suggestions to improve it.

**Comments:**

**Introduction**

1. The introduction section is very short and appears to jump between different studies and concepts without clear transitions, which can disrupt the logical flow. Try to improve the introduction section.
2. Provide an explicit statement of the objective(s) or question(s) the review addresses.
3. I recommend shortly presenting the structure of the rest of the paper. It helps the reader know what to expect from the rest of the manuscript.

**Literature Review**

1. It would be nice if you could delete this section and discuss the related literature reviews in the introduction section.

**Methodology**

1. Section 3.1. Scopus was selected as the primary source of data due to its extensive indexing of peer-reviewed literature and robust analytical capabilities, but limits the findings generalizability. I recommend to consider other research databases.
2. As discussed in Section 3.2, articles published within the years (2019–2024) have been considered. The use of latest references published in 2024 onwards are limited in your manuscript which is difficult to understand the state of art and current knowledge gap. A comprehensive review of latest, related studies is needed. How does it advance the existing discourse beyond these foundational papers? The authors must take into account the latest development in organizational performance determinants, e.g. Artificial intelligence and other emerging technologies to show their up-to-date knowledge about the matter.
3. Section 3.3. Provide a complete searching strategy/algorithm.
4. I recommend you to provide author keyword co-occurrence map, with network visualization, for the database.
5. Section 3.4. As mentioned in Section 3.4, the review was conducted in accordance with the guidelines set by the Preferred Reporting Items for Systematic Reviews and Meta-Analyses (PRISMA). If so, kindly address the following issues to enhance the impact of your manuscript.

1. Describe methods used to assess certainty/confidence in the evidence for each outcome.
2. Present the methods employed to assess the risk of bias for each included study.
3. Specify effect measures (e.g., risk ratio, mean difference) for each outcome in synthesis/results.
4. Describe synthesis methods, their rationale, and for meta-analyses, detail models, heterogeneity assessment methods, and software.
5. Describe sensitivity analyses performed to assess result robustness.

**Results and Discussion**

1. Section 4. You have said 20 empirical research papers were selected. Empirical research, has limitations such as in generalizing findings, and the possibility of researcher bias or errors. How was this rectified? Hence how do you validate the research findings?
2. Some paragraphs are very short, necessitates detail explanation.
3. Revise Figure 4. Collect and categorize list of publications by subject area, and provide much discussion about the figure.
4. The discussion is lacking of clear definition and direction. The discussion is only reiteration of facts from references and lack of critical review. This not in line with the premise of the paper:
5. Revise the discussion. The comparisons of your findings with previous studies and existing theories are limited. How do your results confirm, contradict, or extend the reviewed literature?

**Conclusion**

1. Section 6 and 7. Conclusion can be put in separate section (last section).

Are the rationale for, and objectives of, the Systematic Review clearly stated?

Yes

Is the statistical analysis and its interpretation appropriate?

I cannot comment. A qualified statistician is required.

If this is a Living Systematic Review, is the ‘living’ method appropriate and is the search schedule clearly defined and justified? (‘Living Systematic Review’ or a variation of this term should be included in the title.)

Partly

Are sufficient details of the methods and analysis provided to allow replication by others?

Partly

Are the conclusions drawn adequately supported by the results presented in the review?

Yes

Reviewer Expertise:

Organizational Performance, AI, Human Resource Management

I confirm that I have read this submission and believe that I have an appropriate level of expertise to confirm that it is of an acceptable scientific standard, however I have significant reservations, as outlined above.

# Reviewer report: Reviewer response for version 1

**DOI:** 10.5256/f1000research.185871.r429242

**Published:** 2025-12-18

**Authors:** Muhammad Khairul Anwar

**Affiliations:** 1 Universitas Negeri Surabaya, Surabaya, East Java, Indonesia

Dear Authors,

 The manuscript is very neatly structured following the PRISMA steps, clearly showing the identification, screening, eligibility, and inclusion sections accompanied by the PRISMA Diagram flow. This demonstrates strong methodological compliance and helps readers assess the transparency of the article selection process. The theme of sustainable organizational performance, combined with PRISMA-based SLR and bibliometrics, is a relevant and current topic. The focus on variables such as knowledge management, sustainable development, digital transformation, and HRM demonstrates good multidisciplinary integration. Its novelty is evident in the effort to link these variables within the framework of sustainability and digital technology. However, I have several suggestions for improvement for your research, as follows:

- I have seen that the inclusion criteria have been set, but I did not find the exclusion criteria, so my suggestion is to add the inclusion criteria.
- Please expand section “3.1. Source.” Please explain in detail.
- This study uses Scopus data sources, which should be explained in the limitations to strengthen the transparency of the article selection process.
- I see that you used several keywords in selecting articles; this should be stated in the limitations.
- Please note Table 1, in the “title” column, I suggest putting quotation marks around each article title you mention. And don't forget to italicize them.
- The discussion has been described and summarizes the findings of the article.
- Please expand on the theoretical and practical implications.

Good Luck

Are the rationale for, and objectives of, the Systematic Review clearly stated?

Yes

Is the statistical analysis and its interpretation appropriate?

Not applicable

If this is a Living Systematic Review, is the ‘living’ method appropriate and is the search schedule clearly defined and justified? (‘Living Systematic Review’ or a variation of this term should be included in the title.)

Not applicable

Are sufficient details of the methods and analysis provided to allow replication by others?

Partly

Are the conclusions drawn adequately supported by the results presented in the review?

Yes

Reviewer Expertise:

Public Administration, Organizational Public, Governance and Organizational Behaviour

I confirm that I have read this submission and believe that I have an appropriate level of expertise to confirm that it is of an acceptable scientific standard, however I have significant reservations, as outlined above.

# Reviewer report: Reviewer response for version 1

**DOI:** 10.5256/f1000research.185871.r429243

**Published:** 2025-11-25

**Authors:** Eka Desy Purnama

**Affiliations:** 1 Krida Wacana Christian University, Jakarta, Jakarta, Indonesia

This article is a well-structured and relevant PRISMA-based systematic review on the theme of sustainable organizational performance.

- The rationale and objectives of the study are well explained in the Abstract and Introduction sections. The reasons for choosing the PRISMA method are clearly stated, namely to ensure transparency and high replication in systematic reviews. The objectives of the study (to identify factors that comprehensively influence organizational performance) appear consistent with the issues raised.
- The research method was systematically developed following the PRISMA steps (identification, screening, eligibility, inclusion). The use of the Scopus database and inclusion-exclusion criteria (2019–2024, English-language articles, peer-reviewed, etc.) were appropriate.
- The conclusions drawn are consistent with the evidence presented, and the practical implications are quite strong.
- The discussion would be more comprehensive if accompanied by a conceptual model summarizing the interrelationships between factors.

Overall, this manuscript is acceptable, especially in terms of the methods and presentation of results, which other researchers can easily replicate. However, I suggest adding exclusion criteria to increase the transparency of the article selection process.

Are the rationale for, and objectives of, the Systematic Review clearly stated?

Yes

Is the statistical analysis and its interpretation appropriate?

Not applicable

If this is a Living Systematic Review, is the ‘living’ method appropriate and is the search schedule clearly defined and justified? (‘Living Systematic Review’ or a variation of this term should be included in the title.)

Not applicable

Are sufficient details of the methods and analysis provided to allow replication by others?

Yes

Are the conclusions drawn adequately supported by the results presented in the review?

Yes

Reviewer Expertise:

Management

I confirm that I have read this submission and believe that I have an appropriate level of expertise to confirm that it is of an acceptable scientific standard.
